# In-process analytics for IVT mRNA manufacturing: from process understanding to advanced process control

**DOI:** 10.3389/fbioe.2026.1800423

**Published:** 2026-03-11

**Authors:** Naryeong Kim, Emily Dong, Kate Tschudi, Julien Camperi

**Affiliations:** Cell Therapy Engineering and Development, Genentech, 1 DNA Way, South San Francisco, CA, United States

**Keywords:** advanced process control, in-process analytics, IVT mRNA, process analytical technology (PAT), process understanding

## Abstract

RNA therapeutics are expanding rapidly, driving demand for manufacturing processes that can keep pace with clinical translation. Because mRNA yield and impurity profiles are jointly influenced by upstream plasmid DNA (pDNA) preparation and the *in vitro* transcription (IVT) reaction, in-process measurements are increasingly applied across the product lifecycle, albeit with distinct objectives in process development versus current good manufacturing practice (cGMP) production. Within the U.S. Food and Drug Administration’s Process Analytical Technology framework, we categorize in-process analytical methods by control intent: (1) measurements that build process understanding and define operating windows; (2) in-process controls (IPCs) that support predefined stop/go, forward-processing, and endpoint decisions; and (3) measurements that could enable advanced or adaptive process control (APC) through closed-loop feedback. We discuss how each category is deployed during process development and in cGMP manufacturing. Following the workflow from pDNA preparation through IVT, we highlight analytical measurements that establish template readiness—such as plasmid topology, linearization completeness, and co-purifying impurities that can propagate into transcription performance and complicate downstream processing—as well as time-resolved measurements during IVT that track reactant consumption and product formation to inform endpoint selection, feed timing, and deviation triage under predefined decision rules. We compare the strengths, implementation constraints, and validation considerations of at-line, on-line, and in-line approaches, and identify key gaps that currently limit broader adoption, including practical time-resolved quantification of double-stranded RNA and the availability of production-ready in-line sensing technologies. Collectively, these in-process analytics deliver near-term value by enabling process understanding and IPC-based decision support, while establishing the foundation required for future APC in mRNA manufacturing.

## Graphical Abstract

**Figure:**
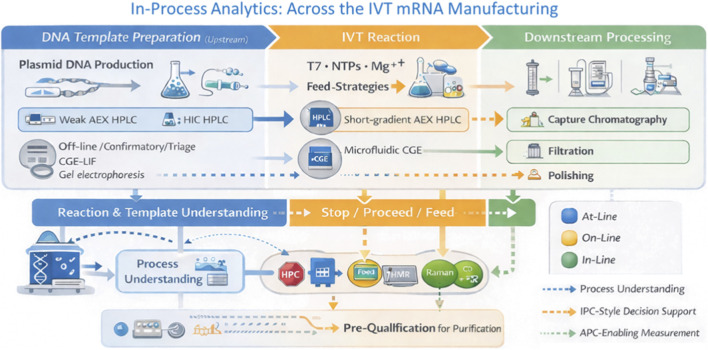

## Introduction

RNA has expanded beyond vaccines into a broad therapeutic class encompassing cancer immunotherapies, protein replacement therapies, and personalized medicines, making scalable and well-controlled manufacturing central to clinical translation (Parhiz et al., 2024; Shi et al., 2024; Żak et al., 2026). *In vitro* transcription (IVT) produces mRNA from DNA templates using T7 RNA polymerase in defined nucleotide and Mg^2+^ buffer systems, often coupled with co-transcriptional capping chemistries (Henderson et al., 2021; Zhang et al., 2023a; Nair and Kis, 2024). IVT performance is governed by substrate availability, magnesium complexation, DNA template quality, and product inhibition by pyrophosphate (Cunningham and Ofengand, 1990). These constraints can be mitigated through enzymatic pyrophosphate removal or optimized feeding strategies that sustain favorable reaction kinetics over time (Tersteeg et al., 2022; Boman et al., 2024). Collectively, these variables shape yield and impurity trajectories—and therefore final mRNA quality—motivating measurement strategies that provide actionable, time-resolved process information. In-process measurements are increasingly deployed across the mRNA manufacturing lifecycle, but their roles differ between process development and current good manufacturing practice (cGMP) production. During development, measurements primarily support process understanding by linking critical process parameters to mRNA quality attributes and defining robust operating windows. In cGMP manufacturing, measurements are more commonly implemented as predefined in-process controls (IPCs) that support stop/go decisions, forward-processing readiness, and endpoint determination, rather than enabling dynamic or adaptive control.

**FIGURE 1:**
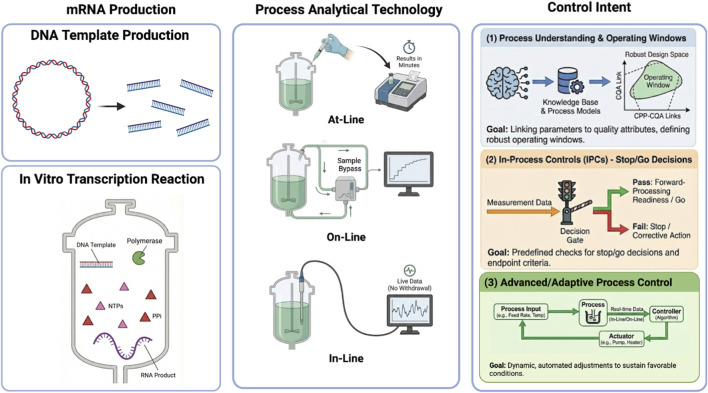
In-process analytics and control intent across DNA template preparation and IVT. The left panel illustrates key upstream stages of mRNA production, including DNA template preparation and the IVT reaction. The center panel summarizes PAT measurement modalities—at-line, on-line, and in-line—which differ by sampling strategy and data latency but are independent of control intent. The right panel categorizes measurements by control intent: (1) process understanding, where measurements support mechanistic insight and definition of robust operating windows; (2) IPCs, where predefined decision rules enable stop/go, forward-processing readiness, and endpoint determination; and (3) APC, where real-time measurements are integrated with automated control algorithms to dynamically adjust process inputs. Most current pDNA and IVT analytics deliver value through process understanding and IPC decision support, while also establishing the measurement foundation required for future APC implementation.

We frame this review within the U.S. Food and Drug Administration’s Process Analytical Technology (PAT) framework, which defines PAT as a system for designing, analyzing, and controlling manufacturing through timely measurements during processing, embedded within a broader strategy for process understanding and control (Food and Drug Administration, 2009). Throughout, we distinguish measurement modality—at-line, on-line, or in-line—from control intent and discuss how analytical methods support (i) process understanding, (ii) IPC-based decision-making, and, in select cases, (iii) measurement layers that could enable advanced or adaptive process control (APC). Our focus is on in-process measurements spanning DNA template preparation and the IVT reaction through handoff into downstream processing (Figure 1).

During IVT, a range of product-related impurities—including double-stranded RNA (dsRNA), fragmented transcripts, DNA-RNA hybrid intermediates, aggregates, and uncapped species—are known to arise and must be controlled to achieve high-purity mRNA products (Camperi et al., 2025; Lenk et al., 2024). These impurities directly influence the complexity and load of subsequent purification operations, such as ion-exchange and reversed-phase chromatography, which are routinely employed to reduce dsRNA and other byproducts (Zhang et al., 2023b; Baiersdörfer et al., 2019). While mRNA purification operations—such as chromatography- and filtration-based capture and polishing steps—also rely on in-process analytics and decision criteria, downstream PAT and IPC strategies are referenced here only insofar as they relate to IVT-derived impurity burden, as the primary focus of this review is on DNA template preparation and IVT-centered process analytics. Accordingly, upstream and IVT-related PAT tools described in this review often shape downstream purification burden and forward-processing readiness. Where relevant, we highlight how upstream and IVT measurements influence downstream purification burden and inform forward-processing readiness decisions (Figure 1).

We use standard PAT terminology to describe monitoring approaches. At-line assays analyze manually withdrawn samples with turnaround times on the order of minutes. Online systems employ automated sampling to provide near-continuous data streams, while in-line sensors interrogate the process directly without sample withdrawal (Food and Drug Administration, 2009). As methods are introduced, we classify them using this terminology and note differences in implementation between process development and cGMP manufacturing contexts.

### In-process methods for linear DNA template production

Linearized DNA template is a critical material for IVT. Typically, linearized DNA is derived from plasmid DNA produced by cultivating engineered *E. coli*, followed by cell harvest, alkaline lysis and neutralization, clarification, and chromatographic operations that remove host-cell proteins, lipids, genomic DNA, and process-related impurities (Whitley et al., 2022a; Martínez et al., 2023; Shankar et al., 2024). Subsequent polishing steps enrich the supercoiled plasmid isoform while reducing open-circular and linear species (Pavlin et al., 2023; Piao et al., 2024). The purified plasmid is then linearized using a restriction enzyme to generate a full-length transcription template. The purity and structural integrity of this linearized material have a measurable impact on impurity formation during the IVT reaction, particularly double-stranded RNA (dsRNA) generation (Martínez et al., 2023).

In practice, template quality assessment establishes predefined “go/no-go” decision points prior to IVT charge, such as accept versus repolish, re-linearize, or hold. Consequently, most pDNA template analytics are implemented as at-line or off-line assays with short turnaround times, rather than as continuous measurements. In-line sensing of pDNA attributes is not yet common, and true on-line pDNA analytics remain rare outside of autosampler-based sampling configurations.

#### At-Line anion exchange chromatography for isoform separation

DNA attributes that most consistently shape mRNA quality include plasmid topology, integrity of the linearized template, and residual impurities carried over from plasmid production. Topology is particularly important because the fraction of supercoiled relative to open-circular material determines the concentration of initiation-competent templates and influences early IVT kinetics, thereby shaping titer trajectories and time to plateau (Kralj et al., 2023; Pavlin et al., 2023; Piao et al., 2024).

To enable at-line characterization of plasmid isoforms, weak anion-exchange chromatography (AEX) methods have been developed using convective monoliths with 6 µm channels (Table 1), achieving baseline separation of supercoiled, open-circular, and linear isoforms for plasmids below approximately 16 kbp using short gradients and minimal sample preparation (Pavlin et al., 2023). For plasmids up to ∼15 kbp on 6 µm diethylaminoethyl (DEAE) monoliths, these methods enable rapid and quantitative separation of topological variants with good resolution between supercoiled and open-circular species (resolution ≥3). However, separation of open circular from linear isoforms requires optimized chaotropic conditions and typically achieves only moderate resolution (>1.0). At the upper size range, quantitative bias and flow-rate sensitivity further constrain analytical qualification for routine use (Pavlin et al., 2023). Beyond monolithic platforms, packed-bed strong and weak anion-exchange columns are frequently employed for the separation of plasmid isoforms. Specific analytical columns, such as the Protein-Pak Hi Res Q (Waters) and the TSKgel DNA-NPR (Tosoh), have been applied to resolve supercoiled, open circular, and linear plasmid species across a variety of plasmid sizes. These methods utilize non-porous resin (NPR) technology to minimize mass transfer limitations and enable the rapid, quantitative separation of topological variants. For plasmids up to ∼15 kbp, these columns can achieve high resolution (resolution >3) between supercoiled and open-circular species, though the separation of open-circular from linear isoforms often requires optimized chaotropic conditions to achieve moderate resolution.

**TABLE 1:** Summary of in-process analytical methods for plasmid DNA and IVT monitoring**.** Assays are categorized by PAT type, analytical scope, and decision impact, with representative strengths and limitations for each method. The table illustrates how complementary at-line, on-line, and in-line tools collectively provide real-time visibility and control across the mRNA production workflow.

Stage	Assay	PAT category	What it measures	Readout	TAT	Control Intent	Decision impact	Advantages	Limitations	Ref.
pDNA	AEX-HPLC isoform profiling	At-line	Supercoiled, open-circular, linear fractions	AEX-UV peaks for isoforms	About 4–8 min/sample	Builds process understanding of template topology/linearization effects on mRNA quality and supports IPC template-readiness gating, but not APC-enabling because it is discrete/at-line	Determine initiation-competent templates, accept or rework lots	Fast, topology resolved, small aliquots needed	Method tuning may be necessary, as the procedure’s dependency is linked to the IVT outcome	Kralj et al., 2023; Pavlin et al., 2023; Piao et al., 2024
pDNA	HIC screen for co-contaminants	At-line or near-line	Hydrophobic burdens such as host proteins, lipids, and reagents	HIC-UV profiles	About 7–10mins/sample	Supports process understanding and IPC triage/readiness criteria for impurity burden, but not typically APC-enabling due to non-continuous, property-based readout	Diagnose upstream drift, verify polishing	Orthogonal to AEX, good for triage	Not topology specific, matrix-dependent interpretation	Diogo et al. (2003)
pDNA	CE	Off-line	High-resolution sizing and small fragments	CE-UV/FL profiles	About 10 min	Primarily a process understanding and confirmatory IPC tool for integrity/fragmentation, and rarely APC-enabling for in-run control due to turnaround and sampling constraints	Investigations and orthogonal method development	Quantitative, high resolution	Off-line, batching limits speed	Wei et al. (2023)
pDNA	Enzymatic digest + AEX-HPLC for residual DNA	At-line	Sequence agnostic template carryover	Nuclease digest, AEX-HPLC-UV profiles	Minutes to under an hour	Supports process understanding of DNA clearance and IPC acceptance checks for residual template, but is not APC-enabling because it is an at-line decision-point assay	Check template removal in IVT materials	Primer free, IVT matrix compatible	Needs validated digest conditions	Leban et al. (2024)
IVT	Short-gradient AEX-HPLC time course	At-line	Intact mRNA, residual DNA, cap analogs, NTPs	Weak AEX-UV of resolved components	About 4–8 min/sample	Enables process understanding via kinetic profiles and supports IPC endpoint/feed decision rules, and can be APC-enabling if automated and validated for feedback use	Time feeds, choose stop time, triage abnormal runs	Quantitative, sequence agnostic, multiplexed readout	Requires sampling and robust short gradients	Welbourne et al. (2024)
IVT	Flow-NMR ^1^H and^31^P	On-line	NTPs, cap analogs, phosphate, pyrophosphate under process conditions	Diverted stream through NMR flow cell with continuous spectra	Seconds to minutes	Strengthens process understanding of low-MW chemistry and supports IPC verification of interventions, while being strongly APC-enabling due to near-continuous state tracking	Verify feed response, manage magnesium and phosphate balance	Label-free, continuous, true reactor state	Specialized hardware, fluidics	Sarkar et al. (2024)
IVT	AEX-HPLC-guided fed-batch control	At-line control scheme	Uses live HPLC data to trigger staged NTP and magnesium feeds	AEX loop with preset thresholds and feed recipes	5–15 min feedback loop	Translates process understanding into IPC feed/stop rules and becomes APC only when those rules are closed-loop automated and validated	Raise yield and space-time productivity, lower dsRNA vs. single shot	Direct and actionable, better enzyme use	Discrete not continuous, needs robust thresholds	Pregeljc et al., 2023; Guo et al., 2024
IVT	Fluorescence RT-IVT assays	Near-line or off-line adjunct	Real-time mRNA formation in small volumes	Fluorophore quencher probes with plate-reader or thermocycler	Seconds to minutes	Mainly process-understanding tools for development screening and are generally not GMP IPC or APC-enabling due to deployment/representativeness constraints	Screen promoters and enzymes, scout kinetics	Very sensitive, very fast, low sample use	Sequence targeted, probe design that rely on sequence-specific molecular sensors, less transferable to manufacturing	Zhang et al., 2023b, Lee et al., 2023
IVT	In-line optical probes Raman or FTIR	In-line emerging	Global chemical trends and selected bands	Immersed probe with chemometric models	Seconds to minutes	Support process understanding via chemometric model building and can serve IPC thresholds, while being APC-enabling when calibrated models provide continuous decision-grade signals	Potential continuous progress tracking and deviation alerts	No sampling, closed systems friendly	Limited specificity without strong models, less proven for IVT	Brauns and Dyer, 2005; Valentini et al., 2022

Abbreviations: AEX, Anion Exchange Chromatography; APC, Advanced or Adaptive Process Control; CE, Capillary Electrophoresis; dsRNA, Double-Stranded RNA; FL, Fluorescence; HIC, Hydrophobic Interaction Chromatography; IPC, In-Process Control; NMR, Nuclear Magnetic Resonance; NTP, Nucleoside Triphosphate; pDNA, Plasmid DNA; Ref. – Reference; RT, Real Time; TAT, Turnaround Time; UV, Ultraviolet.

Because supercoiled fraction directly impacts the quality of the linearized template—and consequently IVT yield and mRNA impurity formation—at-line monitoring of supercoiled, open-circular, and linear area percentages is essential for process control (Piao et al., 2024). A similar short-run weak-AEX method can be applied directly post-linearization to confirm the presence of a single dominant linear species and the absence of residual supercoiled or open-circular material. When chromatographic shoulders or partial co-elution are observed, orthogonal off- or near-line methods such as agarose or polyacrylamide gel electrophoresis, or higher-resolution capillary gel electrophoresis with laser-induced fluorescence (CGE-LIF), can be used to adjudicate template purity and detect small fragments (Loening, 1967; Koontz, 2013; Wang et al., 2022; He et al., 2024). Deployed for same-shift confirmation or rapid near-line triage, CGE-LIF improves discrimination when short-gradient AEX indicates potential co-elution and is particularly useful for verifying post-digest purity prior to IVT charge (Table 1).

From a PAT perspective, weak-AEX isoform profiling is most valuable as (i) a process-understanding tool during development, enabling quantitative linkage between topology distributions, IVT yield, and impurity formation, and (ii) an IPC-style decision-support method that gates template readiness (e.g., proceed versus repolish; proceed versus re-linearize) using predefined criteria and same-shift at-line results. In contrast, these approaches are not well positioned to support true adaptive process control in the pre-IVT pDNA context, as they rely on discrete sampling and chromatographic cycle times. Their control impact is therefore procedural—triggering predefined decisions—rather than continuous, closed-loop adjustment.

#### At-line hydrophobic interaction chromatography

Hydrophobic co-contaminants pose a recurring risk during plasmid DNA (pDNA) manufacture, as crude or intermediate process streams can carry host-cell proteins, denatured nucleic acids, lipids, or lipopolysaccharides (LPS), and residual reagents that are not reliably captured by simple A260/A280 ratios and can distort ion-exchange peak shape and recovery (Diogo et al., 2003; Freitas et al., 2009). To address this gap, Diogo et al. adapted a phenyl hydrophobic-interaction HPLC (HIC-HPLC) method that provides a rapid, property-based assessment of hydrophobic impurities (Table 1). Under their conditions, double-stranded pDNA elutes in the flow-through, followed by sequential elution of impurities with increasing hydrophobicity. The method achieves minutes-scale runtimes (∼7 min) without nuclease pretreatment and demonstrates linear calibration over 0–20 μg mL^-1^, low %RSD, and sensitivity at the low μg mL^-1^ level, enabling application across unit-operation samples to trend impurity clearance (Diogo et al., 2003).

Operationally, the phenyl-HIC assay can be applied directly to crude or intermediate process aliquots with simple salt matching. As an orthogonal complement to weak AEX, it characterizes hydrophobic impurity burden and helps verify the effectiveness of upstream purification steps. Its principal limitation is the lack of topology specificity: supercoiled, open-circular, and linear DNA co-elute in the early pDNA peak. Accordingly, HIC-HPLC is best positioned as a triage or confirmation assay for hydrophobic contaminants, while weak AEX—supplemented by CGE-LIF or gel-based methods when needed—remains the primary approach for isoform-resolved template assessment.

Because true on-line or in-line options for pre-IVT plasmid analytics remain limited, DNA template assessments are typically executed at-line with short turnaround times. In process development, phenyl HIC-HPLC is therefore valuable for trending hydrophobic impurity clearance across unit operations and identifying conditions that correlate with downstream IVT variability. In cGMP manufacturing, it is best positioned as an IPC-style, same-shift check on hydrophobic burden to support predefined disposition decisions (e.g., proceed versus repolish or hold), particularly when co-contaminants are suspected to interfere with ion-exchange analytics or propagate into IVT impurity risk.

#### IVT in-process analytics and control

Within the IVT reactor, yield, cost, and impurity profiles are determined by the dynamic consumption of reactants and accumulation of byproducts. Time-resolved in-process analytics are therefore essential for actionable control and optimization. During process development, these measurements map reaction kinetics, identify dominant levers (e.g., Mg^2+^ and NTP balance), and establish feeding and endpoint rules to maximize yield while managing impurities. In cGMP manufacturing, measurements are most often applied as IPC-style decision support—predefined stop times, feed/hold strategies, or forward-processing readiness—rather than fully adaptive closed-loop control, which remains challenging to validate and implement.

#### At-line anion-exchange HPLC for IVT feeding optimization

Short-gradient, at-line AEX-HPLC illustrates these principles in practice. Traditional reverse-phase HPLC methods require 20–30 min per run, too slow for real-time monitoring. Short-gradient AEX-HPLC achieves ∼4–8 min per injection and resolves intact mRNA, residual DNA template, cap analogs, and all four NTPs (Table 1). This enables quantitative time courses of mRNA formation and NTP consumption in batch or fed-batch operations (Pregeljc et al., 2023; Megušar et al., 2024; Welbourne et al., 2024; Camperi et al., 2025).

AEX-HPLC informs practical control decisions: selecting stop times when mRNA plateaus, triggering or sizing NTP and Mg^2+^ feeds, and flagging runs with unexpected template or cap-analog behavior. Strengths include speed, sequence-agnostic detection, and quantitative accuracy; limitations include maintaining resolution across changing matrices and reliance on small, consistent sample withdrawals at larger scale (Welbourne et al., 2024). Pregeljc et al. demonstrated data-driven feeds that doubled yield (∼10 mg mL^-1^ in 3 h) and enabled at-line capping quantification (79% ARCA cap). While (Pregeljc et al., 2023; Megušar et al., 2024; Welbourne et al., 2024; Camperi et al., 2025) utilized active feedback, (Guo et al., 2024) pivoted to an open-loop strategy, demonstrating that pre-planned three-phase feeding schedules could improve output without the need for at-line analytics, provided that preliminary screens first identify NTP and Mg^2+^ as the dominant levers. Overall, short-gradient AEX-HPLC supports development optimization, IPC-style decisions, and can serve as a measurement layer for advanced process control when integration and validation allow.

#### At-line electrophoretic integrity analytics

CGE and CGE-LIF provide high-resolution analysis of full-length mRNA and fragment distributions, complementing AEX-HPLC. Certain chemistries also resolve poly(A) tails, and studies report linear, precise quantitation suitable for development and QC triage (Martínez et al., 2023; He et al., 2024). Microfluidic chip CE runs in 1–3 min per lane, enabling high-throughput screens and dense time-course studies. These methods flag conditions for confirmatory CGE or AEX analysis rather than driving closed-loop control (Wang et al., 2022; He et al., 2024). Bench-scale Surface Plasmon Resonance (SPR) assays measure polymerase kinetics on immobilized templates, providing mechanistic insight for enzyme and sequence selection upstream of manufacturing (Greive et al., 2008). While at-line assays provide discrete snapshots, continuous trending between injections can be achieved using on-line or in-line spectroscopic methods.

#### On-line and in-line spectroscopic methods

Flow Nuclear Magnetic Resonance (NMR) offers near-continuous tracking of low-molecular-weight species, enabling label-free quantitation of NTPs, cap analogs, inorganic phosphate, and pyrophosphate. It complements AEX-HPLC by confirming feed responses and modeling Mg^2+^–pyrophosphate balance (Sarkar et al., 2024). Adoption is limited by hardware requirements, sampling representativeness, and spectral complexity.

Raman spectroscopy provides a label-free, in-line vibrational fingerprint that can be calibrated to track NTPs and bulk mRNA. Statistical models (PLS, SVR) allow regulatory-grade quantitation, though matrix drift and fluorescence interference must be managed (Barton et al., 2022; Matuszczyk et al., 2023).

Structural measurements—Circular Dichroism (CD) and Time-resolved infrared spectroscopy (TR-IR)—offer insights for construct design. CD reports mRNA secondary structure and base stacking, flagging structural liabilities before scale-up (Sosnick et al., 2000; Le Brun et al., 2020; Widom and Hoeher, 2022), while TR-IR tracks fast folding/unfolding kinetics to optimize folding pathways and avoid kinetic traps (Brauns and Dyer, 2005; Howe et al., 2023).

#### Real-time fluorescence assay methods

Fluorescence-based assays enable rapid, sensitive monitoring of mRNA synthesis. mRNA aptamers bind fluorogenic compounds, giving sub-minute readouts; the Universal Fluorescence Module (UFM) design uses a self-excising hammerhead ribozyme for sequence independence (Paige et al., 2011; Höfer et al., 2013; Dolgosheina et al., 2014, p. 2013, 2014). Fluorescence Resonance Energy Transfer (FRET)-amplified variants increase sensitivity ∼10-fold, though photobleaching remains a consideration (Han et al., 2013; Hyun Lee et al., 2022).

Sequence-specific probes—including antisense, dual-FRET, binary quencher schemes, molecular beacons, and LNA probes—offer detection down to 2 nM (Ishiguro et al., 1996; Sei-Iida, 2000; Nomura and Kinjo, 2004; Niederholtmeyer et al., 2013; Wang et al., 2018; Lee et al., 2023). These are best applied for enzyme/template screening and kinetic mapping, complementing sequence-agnostic PAT methods like AEX-HPLC and NMR, rather than serving as in-line PAT at production scale.

### Enzyme activity monitoring during IVT

Several PAT tools described above can be applied to monitor enzyme activity during IVT, including T7 RNA polymerase, inorganic pyrophosphatase (IPP), and capping enzymes. Real-time fluorescence systems provide kinetic readouts of RNA synthesis for polymerase screening and rate modeling (Paige et al., 2011; Höfer et al., 2013; Dolgosheina et al., 2014). At-line AEX-HPLC quantifies NTP consumption and mRNA formation to assess polymerase performance and reaction efficiency (Pregeljc et al., 2023; Megušar et al., 2024; Welbourne et al., 2024; Camperi et al., 2025). Flow-NMR or chromatographic monitoring of pyrophosphate and phosphate supports evaluation of IPP function and Mg^2+^ balance (Sarkar et al., 2024), while LC-based assays quantify cap incorporation and capping efficiency (Pregeljc et al., 2023; Welbourne et al., 2024). Collectively, these measurements support kinetic understanding, feed and endpoint definition, and Quality-by-Design implementation in IVT manufacturing.

### Interface with downstream mrna purification and monitoring

Because IVT-derived impurity burden—particularly dsRNA, truncated transcripts, uncapped species, and residual template DNA—directly alters chromatographic load, peak resolution, and binding capacity, downstream purification operations such as strong or weak anion-exchange chromatography, reversed-phase chromatography, cellulose-based dsRNA depletion, and tangential flow filtration must often adjust gradient windows, pooling criteria, and buffer conductivity setpoints to accommodate impurity-driven shifts in separation performance (Baiersdörfer et al., 2019; Zhang et al., 2023a; Megušar et al., 2023; Whitley et al., 2022b). In these operations, in-process monitoring typically relies on UV absorbance profiles for peak detection and fractionation decisions, conductivity and pH tracking during gradient control, and pressure trends to assess column or membrane performance. Although several analytical modalities—including UV/Vis detection, Raman spectroscopy, and chromatographic analytics—appear both in IVT monitoring and downstream purification, their implementation and control intent differ substantially. In IVT, these tools are primarily deployed to quantify reaction kinetics, NTP consumption, product formation, and impurity generation for feed and endpoint decisions (Pregeljc et al., 2023; Sarkar et al., 2024; Camperi et al., 2025). In contrast, within downstream purification the same measurement platforms are interpreted through the lens of chromatographic resolution, impurity clearance, and fraction pooling criteria, often under higher salt and load conditions requiring distinct calibration and decision thresholds (Zhang et al., 2023b; Megušar et al., 2023).

## Conclusion

mRNA manufacturing performance is jointly determined by DNA template readiness and IVT kinetics. In-process analytics are impactful only to the extent they enable clear, actionable decisions. This review distinguishes measurement modality (at-line, on-line, in-line) from control intent, clarifying which tools: (i) build process understanding during development, (ii) function as IPC-style decision support in GMP, and (iii) may serve as APC-enabling measurement layers. Currently, the most deployable methods deliver timely, decision-relevant readouts with manageable validation, including at-line weak AEX and phenyl-HIC for template readiness and impurity burden, and minutes-scale at-line AEX-HPLC for IVT reaction monitoring and feeding control. On-line and in-line methods—flow NMR, Raman, chemometrics—enable continuous trending between injections, but fully adaptive closed-loop control remains uncommon. Near-term impact derives from defining robust operating windows and decision criteria, while long-term progress depends on time-resolved dsRNA quantification and production-ready in-line sensing with validated, transferable models.
